# Advanced Glycation End Products as a Potential Target for Restructuring the Ovarian Cancer Microenvironment: A Pilot Study

**DOI:** 10.3390/ijms24129804

**Published:** 2023-06-06

**Authors:** Elizabeth I. Harper, Michael D. Siroky, Tyvette S. Hilliard, Gena M. Dominique, Catherine Hammond, Yueying Liu, Jing Yang, Veronica B. Hubble, Danica J. Walsh, Roberta J. Melander, Christian Melander, Matthew J. Ravosa, M. Sharon Stack

**Affiliations:** 1 Integrated Biomedical Sciences Graduate Program, University of Notre Dame, Notre Dame, IN 46556, USA; 2 Department of Chemistry & Biochemistry, University of Notre Dame, Notre Dame, IN 46556, USA; 3 Harper Cancer Research Institute, University of Notre Dame, Notre Dame, IN 46617, USA; 4 Center for Functional Anatomy and Evolution, Johns Hopkins University School of Medicine, Baltimore, MD 21205, USA

**Keywords:** ovarian cancer, advanced glycation end product (AGE), collagen, omentum, microenvironment, AGE breakers, Alagebrium, ALT-711

## Abstract

Ovarian cancer is the sixth leading cause of cancer-related death in women, and both occurrence and mortality are increased in women over the age of 60. There are documented age-related changes in the ovarian cancer microenvironment that have been shown to create a permissive metastatic niche, including the formation of advanced glycation end products, or AGEs, that form crosslinks between collagen molecules. Small molecules that disrupt AGEs, known as AGE breakers, have been examined in other diseases, but their efficacy in ovarian cancer has not been evaluated. The goal of this pilot study is to target age-related changes in the tumor microenvironment with the long-term aim of improving response to therapy in older patients. Here, we show that AGE breakers have the potential to change the omental collagen structure and modulate the peritoneal immune landscape, suggesting a potential use for AGE breakers in the treatment of ovarian cancer.

## 1. Introduction

Ovarian cancer (OvCa) is the deadliest gynecological malignancy, with a five-year survival rate below 40% [1]. Most patients are diagnosed after the cancer has spread to a distant site, most commonly the omentum. The omentum is an immune organ rich in adipose and collagen that serves as the abdominal filter and is generally removed as part of the initial debulking surgery after metastatic OvCa diagnosis [2]. OvCa is an aging-associated disease, with most women diagnosed over the age of 60, with an average age at diagnosis of 63. Older patients are also more likely to die from their disease [1].

Metastasizing OvCa cells initially home to the omentum, with omental collagen providing structure for the metastatic tumors and adipose tissue providing metabolic support. The omentum is also an immune organ, with “milky spots” composed of macrophages, lymphocytes, and mast cells [3]. There are documented age-related changes in adipose, collagen, and immune populations that can impact OvCa metastasis [4]. Specifically, we have previously shown age-related changes in collagen ultrastructure, as well as the presence of advanced glycation end products (AGEs) in collagen isolated from aged mice [5].

AGEs are non-enzymatic post-translational modifications that occur on several proteins, including collagen. AGEs are formed by the reaction of glucose with a lysine amino group on the collagen molecule, resulting in a Schiff base intermediate. These undergo an Amadori rearrangement and are oxidized to form AGEs, which can then be further modified to form crosslinks between collagen molecules, such as pentosidine or glucosepane [6]. The resulting inter-molecular crosslinks alter collagen structure and function by reducing the efficacy of collagenolytic matrix metalloproteinases (MMPs), thereby creating a more compact collagen structure that can disrupt the collagen meshwork in organs such as the skin and omentum [5,7,8,9,10,11,12]. As a non-enzymatic process, AGEs tend to form on long-lived, low-turnover proteins such as collagen and are present at higher rates in older individuals, as well as diabetic patients who have higher levels of blood glucose [13,14,15,16]. AGE accumulation results in collagen that is more aligned and stiffer than non-crosslinked collagen, which has been shown to increase cancer cell migration [17]. These data suggest that small molecules capable of breaking AGE crosslinks have the potential as adjuvant therapeutics in aged individuals.

Small molecules designed to break crosslinks in vivo have passed FDA phase II clinical trials for the treatment of diabetic heart disease. For example, ALT-711 (Alagebrium) has a reasonable margin of safety with no trends noted in serious adverse events, with gastrointestinal symptoms as the only adverse events reported in the treatment group compared to the placebo (Clinical trials NCT00739687, NCT00557518, NCT00662116, and NCT00089713) [6,18]. High levels of blood glucose due to diabetes result in increased formation of AGE crosslinks and stiffening of the arterial walls [19]. Promising in vivo pre-clinical trials in rats showed a decrease in tail collagen crosslinking and a reduction in arterial stiffness following treatment with ALT-711 [20]. Similar trends were noted in canine models as well [21]. Early clinical trials showed breakage of the AGE crosslinks in arterial collagen resulting in a decrease in stiffness in the arterial wall, successfully improving diastolic function but having no overall effect on blood pressure [22]. However, the company producing the drug declared bankruptcy, resulting in a halt of clinical trials before the completion of phase III [23]. Some recent in vivo trials have expanded the testing of ALT-711 to other age-related diseases, such as atherosclerosis and hypertension, and preliminary studies show promise in slowing breast cancer metastasis [24]. A second compound, designated 2C8, has shown AGE-breaking capability in vitro but has yet to be evaluated in vivo [25,26]. However, neither AGE breaker has been evaluated in OvCa.

AGEs have also been linked to the immune system. The omentum is an immune organ, acting as a filter for the peritoneal cavity, playing an important role in the maintenance of peritoneal homeostasis [27]. AGE receptors, most notably the receptor for advanced glycation end products (RAGE) but also including CD36, SRA-1, and Galectin-3, are expressed on immune cells such as monocytes and macrophages [28]. RAGE signaling has been shown to modulate the immune response to sepsis [29], and the presence of AGEs induces dendritic cell maturation, increases T cell activation by dendritic cells [30], and increases T cell immunogenicity [31]. This upregulation of the immune response by AGEs has been implicated in age-related disease such as atherosclerosis, and likely contributes to the well-documented phenomena of inflammaging, the age-associated increase in global inflammation that affect cancer development and progression [30,32]. Additionally, it has been shown that the presence of AGEs skews macrophage polarization towards the M1 phenotype [33]. Moreover, extracellular matrix ultrastructure can be modified by AGES, potentially modulating the immune response [9,34]. The impact of AGE breakers on omental matrix organization and peritoneal immune response in aged mice is explored in this study.

## 2. Results

**Figure 1:**
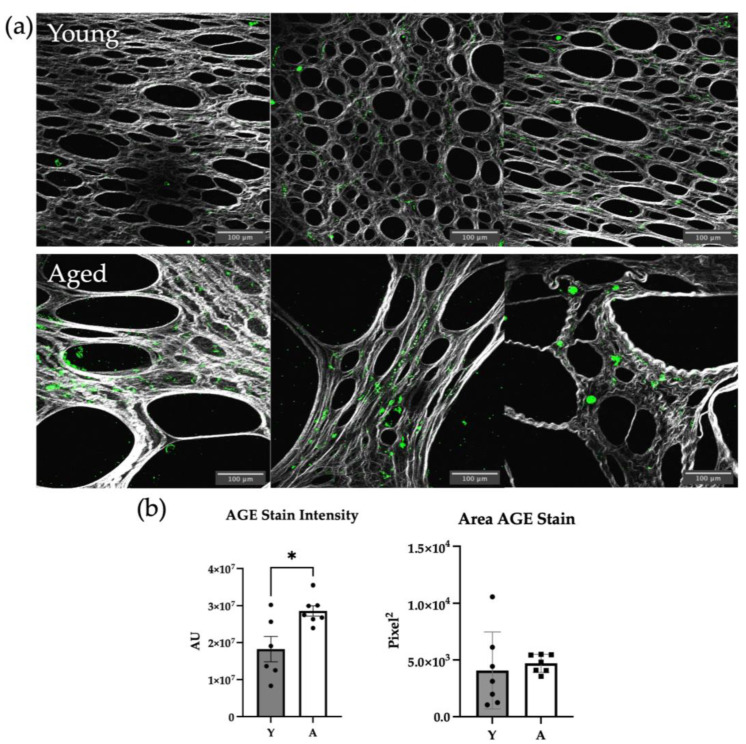
Presence of advanced glycation end products (AGEs) in the omental microenvironment. (**a**) Omenta (n = 7/group) harvested from young (3–6 month) or aged (20–23 month) mice were stained with a fluorescently tagged α-AGE antibody and imaged with a multiphoton microscope in the SHG (collagen, grey) and fluorescent (AGE, green) channels, showing the presence of AGEs in the omental microenvironment. Scale bar 100 μm. (**b**) Quantification of AGE staining showing the average AGE staining intensity per mouse (*p* = 0.0130) and the area stained by the fluorescent AGE antibody (*p* = 0.6430). (* *p* < 0.05).

AGEs crosslink collagen and have been shown to block collagenolysis [7]. We have previously observed an increase in AGE content in isolated aged murine tail collagen relative to collagen from young mice, but omental collagen has not yet been investigated [5]. To examine AGE content in the intact omental tissue, second harmonic generation (SHG) imaging in conjunction with 2-photon excitation fluorescence (TPEF) microscopy using anti-AGE antibodies was employed. AGE staining was observed in both aged and young omental collagen, with a trend toward enhanced AGE staining in aged omenta relative to young (Figure 1a, compare lower to upper panel). Staining intensity was significantly higher in the aged cohort as compared to the young (Figure 1b), while no differences in the overall stain area were observed.

**Figure 2:**
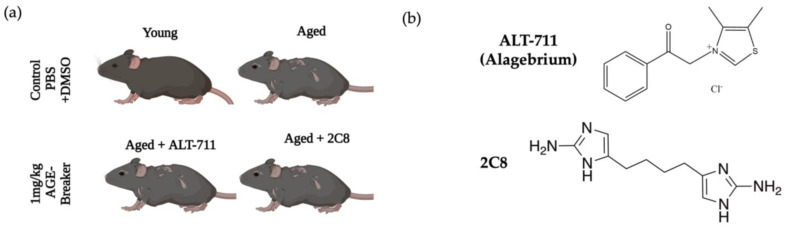
Experimental design. (**a**) Female mice (young: 3–6 month or aged: 20–23 month) in the control group were given an I.P. injection 5 days/week for 4 weeks of the vehicle (0.8 µm DMSO in PBS). The treatment groups (aged: 20–23 months) were given 1 mg/kg of either ALT-711 or 2C8 in 0.8 µM DMSO in PBS injected I.P. 5 days/week for four weeks. Image made with BioRender. (**b**) Chemical structures of AGE breakers ALT-711 (also known as Alagebrium) and 2C8. ALT-711 was chosen as it is the industry standard for AGE breakers and has been shown to be safe and effective in FDA clinical trials, and has been shown to have some anti-cancer effects in breast cancer models [22,24]. 2C8 was chosen as it has been shown to have increased AGE-breaking capability as compared to ALT-711 in vitro, but has not been evaluated in vivo [25,26].

We have previously demonstrated that omental collagen from aged mice displayed significantly enhanced anisotropy with an increase in long linear fibers and thick banding relative to omenta from young mice [5], and similar results are seen in Figure 1a. To evaluate the potential impact of AGE breakers on omental ultrastructure and function, young (Y, 3–6 month) or aged (A, 20–23 month) mice were placed into one of four cohorts (Figure 2a): young + vehicle control, aged + vehicle control, aged + ALT-711, or aged + 2C8. Vehicle control mice were treated 5 days/week with PBS/DMSO via intraperitoneal (I.P.) injection for four weeks. AGE breaker-treated mice (ALT-711 or 2C8; Figure 2b) were treated 5 days/week with 1mg/kg compound in PBS/DMSO via I.P. injection for four weeks.

**Figure 3:**
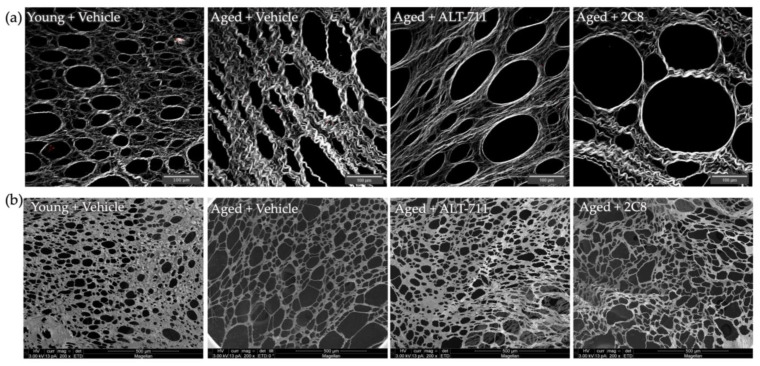
Analysis of AGE breaker-treated omental collagen and structure. (**a**) Omenta (n = 6–7/group) were imaged ex vivo using second harmonic generation (SHG) imaging. Scale bar 100 μm. (**b**) Omenta (n = 6–7/group) were fixed in 2% OsO_4_ and dehydrated, mounted on stubs with carbon stickers, silver painted, and coated with iridium, then imaged using scanning electron microscopy (SEM). Scale bar 500 μm.

To evaluate potential changes in omental collagen ultrastructure in mice receiving AGE breaker treatment, omenta were imaged using SHG to visualize collagen structure. In this cohort, the thick banding of collagen is apparent in the “aged+vehicle” cohort relative to the “young+vehicle” (Figure 3a) [5]. Visual examination shows that the 2C8-treated mice more closely resembled the aged control cohort, with remaining thick banding (Figure 3a). The ALT-711-treated cohort has some structural changes that result in the loosening of the collagen and some restoration of the collagen meshwork (Figure 3a). Importantly, the thick banding of collagen that is characteristic of the aged is visually reduced in the ALT-711-treated cohort.

We have previously observed changes in omental fenestration, with enhanced fenestration area in aged omenta relative to young, and similar results are observed when comparing the “aged+vehicle” cohort to the “young+vehicle” cohort (Figure 3b) [5]. To evaluate the effect of AGE breaker treatment, omenta from the cohorts shown in Figure 2 were fixed and processed for scanning electron microscopy (SEM). This imaging allows visualization of the omentum as a whole organ, not just the underlying collagen matrix.

SHG and SEM data were evaluated using multivariate statistical analyses. Discriminant function analyses (DFA) based on three variables indicate significant differences in the degree of classification among treatment groups. In young (Y) mice, five of six omenta (83%) were classified correctly, with one subject misclassified as aged-vehicle (A-V, Table 1). In A-V, four of six omenta (67%) were identified correctly; one subject was misclassified as Y and one as aged+2C8 (A-2C8). In aged+ALT (A-ALT)-treated mice, two specimens (29%) were classified appropriately, while three subjects were grouped as A-2C8. Interestingly, two A-ALT-treated subjects were classified as Y, indicative of the recovery of a young omental phenotype. In the A-2C8-treated group, three specimens were identified correctly, whereas two were misclassified as A-C and a third as A-ALT.

Additional DFA analyses based on two variables indicate significant differences in the degree of classification among treatment groups. In Y mice, five of six omenta (83%) were classified correctly, with one subject misclassified as A-V (Table 2). In A-V mice, three of six omenta (50%) were identified correctly; two subjects were misclassified as Y, whereas the other specimen was misidentified as A-ALT. In A-ALT-treated mice, two omenta (29%) were classified appropriately, while four subjects were grouped as A-2C8-treated, and one was misidentified as A-V. In A-2C8-treated mice, two specimens were identified correctly, whereas two were misclassified as A-ALT-treated. Interestingly, two A-2C8-treated subjects were classified as Y, indicative of the recovery of a young omental phenotype.

**Figure 4:**
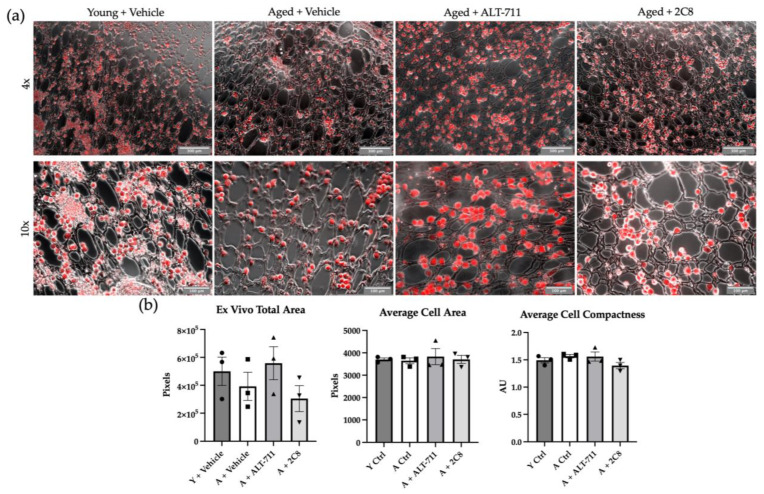
OvCa cell adhesion. (**a**) Omental explants (n = 3/group) were incubated with 10^6^ RFP-tagged ID8*^Trp^*^53-/-^ cells for 2 h prior to gentle washing and imaging. Scale bars denote 300 μm (4×) and 100 μm (10×) (**b**) RFP signal was quantified to analyze adhesion, shown as total area of RFP signal. Average cell area and average cell compactness were measured using Cell Profiler. *p* values listed in Table A1 in Appendix A.

As metastasizing OvCa cells avidly home to the omentum, we next used a three-dimensional explant model to evaluate the adhesion of OvCa cells to omental explants ex vivo. Omenta were removed from control and treated mice immediately following sacrifice, pinned to optically clear silastic resin-coated plates, and used as an adhesive substratum for OvCa cells tagged with a red fluorescent protein (RFP). Two hours after the addition of cells, omenta were carefully washed, imaged, and the RFP signal was quantified to determine cell adhesion (Figure 4). No significant differences were observed in initial omental adhesion between the groups (Figure 4).

**Figure 5:**
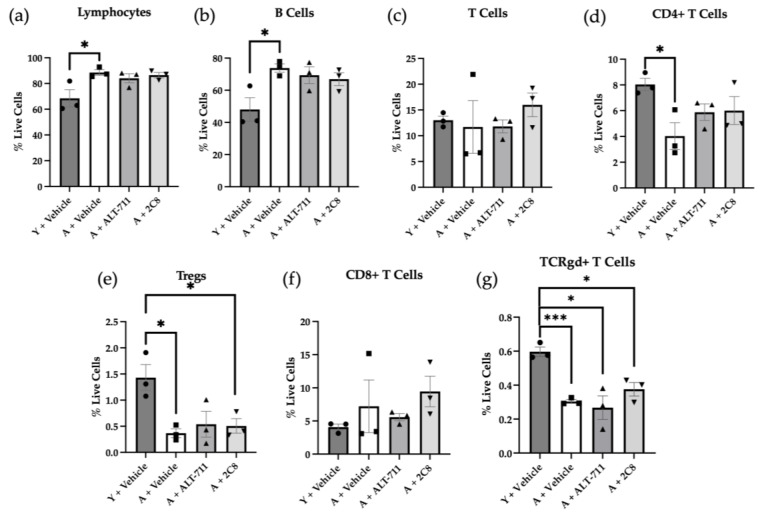
Analysis of peritoneal lymphocyte immune populations. Mice (n = 3/group) were sacrificed, and peritoneal immune cells were harvested via peritoneal lavage and stained for multiplex flow cytometry. Immune types stained for included (**a**) total lymphocytes, (**b**) B cells, (**c**) total T cells, (**d**) CD4+ T cells, (**e**) regulatory T cells (Tregs), (**f**) CD8+ T cells, and (**g**) TCRγδ+ T cells. Student’s *t*-test determined significance (* *p* < 0.05 and *** *p* < 0.0005). *p* values listed in Table A1 in Appendix A. Antibodies are listed in Table A2 and Table A3 in Appendix A.

To examine the influence of AGE breakers on the peritoneal immune landscape, immune cells were harvested by peritoneal lavage, processed, and evaluated using multiplex flow cytometry with a panel of immune cell markers. Results in Figure 5 show the lymphocyte panel. Total lymphocytes were significantly elevated in the “aged+vehicle” and the “aged+2C8” cohort relative to the “young+vehicle” (Figure 5a) that was not observed in the ALT-711 or 2C8 treatment groups. A similar trend was seen in B cells, where the aged control was significantly elevated as compared to the young control, but total cell numbers were not significantly different in the treatment groups (Figure 5b). No changes in total T cells were detected (Figure 5c); however, there was a significant reduction in CD4+ T cells in the aged control group as compared to the young, but again, we see a modest restoration toward the young with the treatment groups (Figure 5d). Tregs were significantly reduced in both the aged control and the 2C8 groups as compared to the young, but not the ALT-711 treatment group (Figure 5e). In contrast, cytotoxic T cells were slightly enhanced, however not significantly, in the aged cohorts (Figure 5f). There was also a noted decrease in TCRγδ+ T cells, or T cells with noncanonical T cell receptors, in the aged cohorts as compared to the young that seemed relatively unaffected by AGE breaker treatment (Figure 5g).

**Figure 6:**
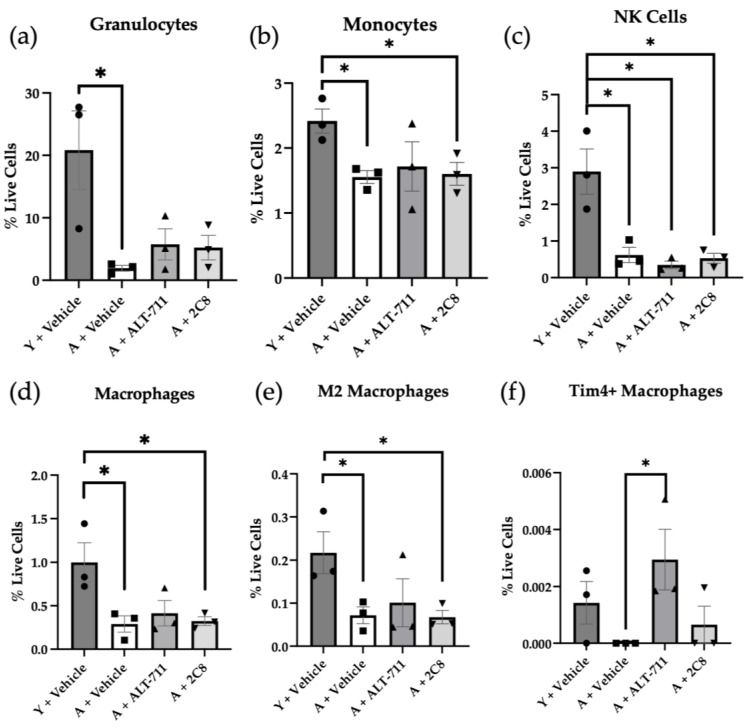
Analysis of peritoneal non-lymphocyte immune populations. Mice (n = 3/group) were sacrificed, and peritoneal immune cells were harvested via peritoneal lavage and stained for multiplex flow cytometry. Immune types stained for included (**a**) granulocytes, (**b**) monocytes, (**c**) natural killer (NK) cells, (**d**) macrophages, (**e**) M2 macrophages, and (**f**) Tim4+ tissue-resident macrophages. Student’s *t*-test determined significance (* *p* < 0.05). *p* values listed in Table A1 in Appendix A. Antibodies are listed in Table A2 and Table A3 in Appendix A.

Results from the analysis of non-lymphocyte immune cell populations are shown in Figure 6. Granulocyte populations were decreased with age, but the only statistically significant difference was observed between the young and aged controls, suggesting a modest restoration with the AGE breaker-treated cohorts (Figure 6a). In monocytes, we saw a statistically significant decrease in the aged control and 2C8-treated cohorts as compared to the young, while the ALT-711-treated cohort was not significantly different from the young control (Figure 6b). In natural killer (NK) cells, there was a significant decrease in all the aged cohorts as compared to the young control (Figure 6c). As seen in total monocytes, macrophages only showed significant changes with the aged control and 2C8-treated groups as compared to the young control (Figure 6d). As reported previously, AGEs tend to skew macrophages toward M1 polarization [33], and this is supported by the modest restoration in M2 macrophages in the ALT-711-treated cohort (Figure 6e). Additionally, the ALT-711-treated group had a significant increase in Tim4+ macrophages, tissue-specific macrophages from the peritoneal cavity, as compared to the aged control group (Figure 6f).

## 3. Discussion

While the presence of AGEs has been well documented in other collagen-rich tissues such as the skin, AGEs in the peritoneal cavity, and specifically in omental collagen, have not been extensively studied. Using immunofluorescence microscopy, our results show that AGE staining intensity is enhanced in omenta from aged mice relative to young. The staining pattern may reflect AGE modification of collagen as well as collagen-bound cells. Collagen is a long-lived protein with a half-life of 15 years or greater [13]. Of note, many cell types have been shown to be AGE-modified [13,35,36,37,38]. The use of alternative methods of tissue preparation, involving fixation and permeabilization, may enable better penetration of AGE antibodies through the mesothelial cell layer to access the underlying collagen in the intact tissue context. Attempts to extract omental collagen for Western blotting analysis were unsuccessful. Nevertheless, our data support the presence of AGE modifications in the omental microenvironment.

However, while changes seen were modest, the observed trend in ex vivo adhesion wherein the ALT-711-treated mice more closely reflected the young control rather than the aged may be indicative of AGE breaking. AGEs have been shown to modify the arginine on the α2β1 integrin-binding motif (GFOGER), thus blocking integrin-mediated cell adhesion [6]. OvCa cells have been shown to rely on α2β1 integrins to bind collagen early in metastatic dissemination, providing a potential mechanism with which to interpret higher levels of adhesion in the young and ALT-711-treated cohorts in an ex vivo setting, where there are few other microenvironmental factors impacting adhesion [39]. This result is in contrast to previous results from our laboratory, wherein we investigated short-term adhesion in vivo and reported enhanced adhesion in aged mice [40]. Several factors could explain this apparent discrepancy, including the time of adhesion (2 h in the ex vivo assay vs. 24 h in the in vivo setting). Additional factors, such as changes in AGE modifications of peritoneal resident immune cells and adipocytes, may also influence in vivo adhesion. Previously, we have shown enhanced omental fenestration in aged mice, which facilitates rapid tumor growth in vivo; however, this same feature may also limit the adhesive surface area in the short-term ex vivo setting [5]. Regardless of initial adhesion kinetics, aged mice have a significantly higher tumor burden than young ones with time [5,40].

To test the hypothesis that treatment with AGE breakers may impact the ultrastructure of aged collagen, SHG imaging was used to investigate omental collagen parameters in intact tissues, and SEM was used to examine omental fenestration. Results were highly variable, showing a range of efficacy across mice, as expected with an aged population where the omental samples tend to be more heterogeneous than those from young mice [5]. Multivariate discriminant function analyses identified two aged mice in the A-ALT-treated cohort and two in the A-2C8-treated cohort that classified with Y mice, indicating that future studies with larger cohorts are warranted. Additionally, the use of a higher dose and/or a longer treatment period with ALT-711 and 2C8 is justified based on the results of this pilot study and the lack of toxicity in treated mice. This is the first report of a study using 2C8, a compound that previously showed promising results in vitro [26], in an in vivo study with live mice.

Evaluation of age-related changes in the peritoneal immune landscape revealed alterations in several cell populations. Specifically, significant age-related increases in total lymphocyte and B cell populations and significant age-related decreases in granulocyte and CD4+ T cell populations were seen between the young and aged control cohorts; however, a modest restoration toward the young cohort was seen in the AGE breaker-treated cohorts. There were also significant age-related decreases in Tregs, total monocytes, macrophages, and M2 macrophages in both the aged control and 2C8 groups as compared to the young, with the ALT-711-treated group showing modest restoration toward the young group in these cell types. While AGEs have been shown to skew macrophages toward an M1 phenotype, which is seen in our modest restoration of M2 populations with ALT-711 treatment, together, these data support the novel observation that AGEs contribute to the regulation of the peritoneal immune landscape in aged mice. However, it is unclear how this would have an overall effect on OvCa metastatic success, as M2 macrophages are generally immunoregulatory and can help the tumor evade immune regulation, and a high M1/M2 macrophage ratio is associated with higher rates of survival in ovarian cancer patients [41]. However, designating M2 macrophages as “bad” or M1 macrophages as “good” is an oversimplification, and further research into macrophage subsets is warranted. For example, M2 macrophages can be further differentiated into four subsets: M2a, which are involved in tissue repair and wound healing; M2 b and M2c, which are involved in immunoregulation and suppression; and M2d, which more directly affect and support cancer progression, such as through angiogenesis [42]. Future analysis of these M2 subpopulations is warranted. Interestingly, our data show a significant increase in Tim4+ macrophages with ALT-711 AGE breaker treatment. Tim4 is a phosphatidylserine receptor in the T-cell immunoglobulin and mucin domain-containing protein (TIM) family [43,44]. Tim4+ macrophages are tissue-specific macrophages resident to the peritoneal cavity, particularly the omentum, that are immunoregulatory. They have been shown to negatively affect CD8+ T cell anti-tumor immunity; however, Tim4 is not expressed by tumor-associated macrophages (TAMs), only tissue-resident macrophages in the microenvironment [45]. Other studies have shown that depleting Tim4+ cells decreases the progression and metastasis of OvCa; therefore, the noted increase with ALT-711 treatment should be kept under consideration when assessing its therapeutic potential [46]. However, this increase may not be sustained, nor is it necessarily indicative of populations within tissues, as only those cells that are circulating in the peritoneal cavity were collected for analysis in the current study.

Interestingly, Tim4+ macrophages that are also positive for MerTK, a receptor tyrosine kinase in the Tyro-Acl-MerTK (TAM) family, are quite adept at clearing apoptotic cells [43,47]. Tim4 is likely responsible for adhering to the apoptotic cells, but requires MerTK to engulf the cell [43]. While macrophages have been shown to express an AGE receptor that identifies longer-lived cells that have been AGE-modified for phagocytosis, previously, there has been no connection identified between AGEs and Tim4 [35]. Other resident tissue macrophages have been shown to intake AGEs via phagocytosis but cannot break them down, resulting in an accumulation of AGEs in the macrophages that reduces phagocytic activity [48]. Therefore, the increase in Tim4+ macrophages we see with ALT-711 treatment could be due to increased circulation through the peritoneal cavity available for harvest by peritoneal lavage, as opposed to greater numbers of Tim4+ macrophages overall. This increase in circulation could be due to the increased availability of macrophages as fewer cells are marked for phagocytosis due to AGE breaker activity, or the breaking of collagen crosslinks by the AGE breaker, releasing the macrophages into the surrounding tissues.

In summary, this pilot study showed the potential for AGE-breaking compounds to modify the OvCa peritoneal metastatic microenvironment in aged mice. Both ALT-711 and 2C8 have demonstrated efficacy in vitro, and neither showed toxicity during the duration of the study; therefore, future studies assessing collagen structure and immune populations after longer treatment periods and/or higher dosages are warranted. Moreover, a long-term study evaluating OvCa metastatic growth in treated mice would provide additional insight into the therapeutic potential of AGE breakers. In conclusion, the use of AGE-breaking compounds in the treatment of OvCa is an avenue worth pursuing. In particular, we have identified a link between AGEs and the peritoneal immune landscape, in particular Tim4+ resident tissue macrophages, that is worthy of further investigation.

## 4. Materials and Methods

Murine models: Cohorts of C57Bl/6 mice (Jackson Labs) were aged to either 3–6 months of age (young, Y; equivalent to women 20–30 years of age) or 20–23 months (aged, A; equivalent to women 60–67 years of age) [49]. All animal procedures were carried out according to the regulations of the Institutional Animal Care and Use Committee at the University of Notre Dame.

Treatment with advanced glycation end product (AGE) crosslink-breaking compounds in vivo: AGE breaker ALT-711, or Alagebrium [22], was synthesized as follows: equimolar (4 mmol) amounts of 2-chloro-acetophenone and 4,5-dimethylthiazole were added to a round-bottom flask with Acetonitrile as a solvent (40 mL, 0.1 M). The reaction mixture was refluxed for 48 h, where the product precipitated out of the hot reaction mixture. After reflux, the reaction mixture was filtered and washed with a 3:7 mixture of absolute ethanol and tert-butyl methyl ether. NMR analysis showed no presence of starting material after workup. The experimental AGE-breaking compound 2C8 was developed and synthesized by the lab of Dr. Christian Melander at the University of Notre Dame, as previously described [25]. AGE breakers or vehicle, 0.8 uM dimethyl sulfoxide (DMSO) in 1× PBS, were injected intraperitoneally (I.P.) into young or aged mice 5 days/week for 4 weeks at a dose of 1 mg/kg, then mice were sacrificed and imaged as described below.

Second harmonic generation (SHG) imaging and analysis: To visualize collagen structure and AGE content, omenta were stained with an α-AGE antibody (6.5 µg/mouse, ab23722, Abcam, Cambridge, MA, USA) which had Alexa Fluor 647 conjugated via Lightning-Link (ab269823, Abcam) and imaged with SHG to detect collagen and TPEF to detect the AGE antibody. To visualize changes to omental collagen structure due to AGE breaker treatment, SHG imaging was used. Omenta from young (n = 6) or aged controls (n = 6), aged + ALT-711 (n = 7), and aged + 2C8 (n = 6) were harvested following sacrifice. For all experiments, tissues were cleaned with PBS and placed onto 22 × 50 coverslips for imaging. Tissues were imaged on an Olympus FV1000 2-photon confocal microscope with XPLN 25× water objective. Organs were imaged in 3D in 1 µm steps with the 860 laser with RXD1 emission filter. Anisotropic analysis was performed on the images with the FibrilTool plugin in ImageJ, as described by Boudaoud et al. [50]. Total collagen and signal intensity were measured in ImageJ (version 1.53 s, NIH) with the analyze particles function to measure area and raw integrated density, respectively [51].

Scanning electron microscopy (SEM) imaging and analysis: To visualize structural changes due to AGE breaker treatment, omenta from young (n = 6) or aged controls (n = 6), aged + ALT-711 (n = 7), and aged + 2C8 (n = 6) were imaged using SEM. Samples were incubated in pre-fixative solution (2% glutaraldehyde and 2% paraformaldehyde in 0.1M Cacodylate buffer, pH 7.3) overnight at 4 °C, then fixed in 1% OsO_4_ and dehydrated with ascending amounts of EtOH as described [52]. Samples were dried in a critical point dryer (Tousimis 931), then mounted on stubs with carbon stickers, silver painted, and sputter-coated with iridium at a thickness of 5 nm. Samples were imaged on a FESEM: Magellan 400 (FEI). Images of young and aged omenta were analyzed in ImageJ using the “analyze particles” function to determine fenestration area percentage and size after thresholding and despeckling of the images.

Ex vivo adhesion: To assess adhesion of human OvCa cells to omenta ex vivo, mice (n = 3/group) were sacrificed, and omenta were harvested and pinned to silicon-coated cell culture plates as described [51]. Omenta were incubated with 1 × 10^6^ RFP-tagged OVCAR8 cells for two hours; then, omenta were rinsed 3 times with cold PBS and imaged for RFP signal using the ECHO Revolve Microscope. RFP signal was quantified using ImageJ, and cell area and compactness were quantified using Cell Profiler.

Collection of peritoneal immune cells by peritoneal lavage: Mice (n = 3/group) were sacrificed by CO_2_ anesthesia followed by cervical dislocation, and peritoneal immune cells of AGE breaker-treated and control mice were harvested by peritoneal lavage immediately following sacrifice. Briefly, skin was resected, and 6 mL of 1x PBS was injected into the peritoneal cavity, shaken gently for 1 min, then all fluid present in the peritoneal cavity was harvested and processed for flow cytometry.

Preparation of cells for flow cytometry: Peritoneal lavage samples were centrifuged at 450× *g* for 4 min, and the supernatant was discarded. Pellets were resuspended in Ammonium-Chloride-Potassium (ACK) lysing buffer (A1049201, Gibco, Grand Island, NY, USA) to lyse red blood cells, then neutralized with cold PBS. Cells were washed and resuspended at a concentration of 10^6^ cells/mL in PBS, and 0.25 μL/mL LIVE/DEAD Yellow stain (L34959, Invitrogen, Eugene, OR, USA) was added to each sample and incubated for 30 min protected from light. The samples were washed and resuspended in 10% fetal bovine serum (FBS) in PBS, and 10^6^ cells from each sample were added per well in a 96-well V-bottom dish. Samples were incubated and protected from light with CD16/CD32 antibody (Biolegend Cat#156604) for 15 min to block non-specific binding of immunoglobulins, then the cocktail of fluorescently tagged antibodies was added for 15 min protected from light. Samples were washed 3 times, then resuspended in 20 μL 10% FBS in PBS and run on the Cytek Northern Lights flow cytometer (Cytek NL-2000, Cytek Biosciences, Bethesda, MD, USA) as described below. Antibodies used in the lymphocyte panel are listed in Table A2 in Appendix A, and antibodies for the macrophage panel are listed in Table A2 in Appendix A.

Flow cytometry and analysis: Samples were analyzed using a Cytek NL-2000 with blue and violet lasers with SpectroFlo software. Samples were run with a medium flow rate with a threshold of 50,000 P1 gated events with live unmixing against stored single-stained peritoneal lavage samples. Analysis was completed using FlowJo. Cells were quantified as percentage of total live cells and were determined as significant (* *p* < 0.05, ** *p* < 0.005, *** *p* < 0.0005) by student’s *t*-test.

Statistical analyses: Multivariate analyses of variance were used to determine if a given subject would be correctly identified as belonging to its designated treatment group based on parameters of a similar scale using discriminant function analyses (DFA, Systat version 11.0), thus offering a quantitative determination of the distinctness and diversity among groups [53]. Using a subset of three measures that maximized the available samples (SHG intensity, SHG anisotropy, and SEM fenestration size), DFA was used to characterize the multivariate patterning of omental collagen ultrastructure in untreated aged vehicle controls (A-V) or omenta from mice treated with AGE breakers (A-ALT or A-2C8) relative to omenta from untreated young (Y) mice (Table 1). As the variables differ in scale, a second DFA based only on two percentage variables (SHG area % and SEM area%), representative of the proportion of omental tissue area covered by fenestrated collagen, was also performed to further explore the effects of AGE breaker treatment on patterns of variation in omental ultrastructure (Table 2).

## Figures and Tables

**Table 1:** Classification matrix based on three variables (cases in row categories classified into columns). Variables used: SHG intensity, SHG anisotropy, and SEM fenestration size.

	Y	A-V	A-ALT	A-2C8	%Correct
Y	5	1	0	0	83
A-V	1	4	0	1	67
A-ALT	2	0	2	3	29
A-2C8	0	2	1	3	50
Total	8	7	3	2	56

**Table 2:** Classification matrix based on two variables (cases in row categories classified into columns). Variables used: SHG area percentage and SEM area percentage.

	Y	A-V	A-ALT	A-2C8	%Correct
Y	5	1	0	0	83
A-V	2	3	1	0	50
A-ALT	0	1	2	4	29
A-2C8	2	0	2	2	33
Total	9	5	5	6	48

## Data Availability

All relevant data are contained in the manuscript.

## Appendix A

**Table A1:** *p* values for all presented data. Significant values are notated in bold, and near-significant values are notated in italics.

Data Set	Y Ctrl vs. A Ctrl	Y Ctrl vs. ALT	Y Ctrl vs. 2C8	A Ctrl v ALT	A Ctrl vs. 2C8	ALT vs. 2C8
Ex Vivo Total Area	0.4934	0.7289	0.2283	0.3469	0.5579	0.1674
Average Cell Area	0.6388	0.8858	0.4808	0.4057	0.7327	0.3458
Average Cell Compactness	0.2654	0.5230	0.2730	0.9343	*0.0612*	0.1821
Lymphocytes	**0.0477**	0.1117	*0.0635*	0.3457	0.5416	0.5860
B Cells	**0.0296**	*0.0763*	*0.0874*	0.4851	0.2204	0.7229
Total T Cells	0.8115	0.4631	0.2876	0.9845	0.4859	0.1851
Tregs	**0.0153**	*0.0634*	**0.0312**	0.5479	0.4389	0.9146
CD8+ T Cells	0.4755	0.1044	*0.0846*	0.7028	0.6530	0.1779
TCRγδ+ T Cells	**0.0006**	**0.0116**	**0.0101**	0.6326	0.1551	0.2470
CD4+ T Cells	**0.0244**	*0.0555*	0.1627	0.2027	0.2572	0.9266
Granulocytes	**0.0401**	*0.0897*	*0.0772*	0.2048	0.1767	0.8785
Monocytes	**0.0152**	0.1746	**0.0337**	0.6988	0.8203	0.7979
NK Cells	**0.0250**	**0.0152**	**0.0201**	0.3041	0.7333	0.3474
Macrophages	**0.0431**	*0.0942*	**0.0422**	0.5157	0.7668	0.5921
M2 Macrophages	**0.0496**	0.1911	**0.0422**	0.6478	0.8714	0.5941
Tim4+ Macrophages	0.1314	0.3069	0.6922	**0.0500**	0.3739	0.1396

**Table A2:** Antibodies with their fluorophores for Cytek Northern Lights multiplex flow lymphocyte panel.

Marker	Fluorophore
CD11c	BB515
CD4	BV750
CD19	BV711
FcERIa	BV480
CD11b	PerCP-Cy5.5
CD8	Spark Blue 550
NK-1.1	BV650
CD25	PE
CD127	BV605
CD117	PE-Cy7
CD16	efluor 450
TCRγδ	PerCP-Vio700
CD3	AF488
Ly6C	BV510
Ly6G	BV785

**Table A3:** Antibodies with their fluorophores for Cytek Northern Lights multiplex flow macrophage panel.

Marker	Fluorophore
Tim4	BV711
CD31	BV480
CD45	PerCP-Cy5.5
CD86	BV785
CD206	PE
CD34	PeCy7
F4/80	efluor 450
